# Mobile Phone Chips Reduce Increases in EEG Brain Activity Induced by Mobile Phone-Emitted Electromagnetic Fields

**DOI:** 10.3389/fnins.2018.00190

**Published:** 2018-04-04

**Authors:** Diana Henz, Wolfgang I. Schöllhorn, Burkhard Poeggeler

**Affiliations:** ^1^Institute of Sports Science, Johannes Gutenberg University Mainz, Mainz, Germany; ^2^Johann-Friedrich-Blumenbach-Institute for Zoology and Anthropology, Faculty of Biology and Psychology, Georg-August-University Göttingen, Göttingen, Germany

**Keywords:** mobile phone radiation, mobile phone chips, electroencephalography, electromagnetic fields exposure, attention

## Abstract

Recent neurophysiological studies indicate that exposure to electromagnetic fields (EMFs) generated by mobile phone radiation can exert effects on brain activity. One technical solution to reduce effects of EMFs in mobile phone use is provided in mobile phone chips that are applied to mobile phones or attached to their surfaces. To date, there are no systematical studies on the effects of mobile phone chip application on brain activity and the underlying neural mechanisms. The present study investigated whether mobile phone chips that are applied to mobile phones reduce effects of EMFs emitted by mobile phone radiation on electroencephalographic (EEG) brain activity in a laboratory study. Thirty participants volunteered in the present study. Experimental conditions (mobile phone chip, placebo chip, no chip) were set up in a randomized within-subjects design. Spontaneous EEG was recorded before and after mobile phone exposure for two 2-min sequences at resting conditions. During mobile phone exposure, spontaneous EEG was recorded for 30 min during resting conditions, and 5 min during performance of an attention test (d2-R). Results showed increased activity in the theta, alpha, beta and gamma bands during EMF exposure in the placebo and no chip conditions. Application of the mobile phone chip reduced effects of EMFs on EEG brain activity and attentional performance significantly. Attentional performance level was maintained regarding number of edited characters. Further, a dipole analysis revealed different underlying activation patterns in the chip condition compared to the placebo chip and no chip conditions. Finally, a correlational analysis for the EEG frequency bands and electromagnetic high-frequency (HF) emission showed significant correlations in the placebo chip and no chip condition for the theta, alpha, beta, and gamma bands. In the chip condition, a significant correlation of HF with the theta and alpha bands, but not with the beta and gamma bands was shown. We hypothesize that a reduction of EEG beta and gamma activation constitutes the key neural mechanism in mobile phone chip use that supports the brain to a degree in maintaining its natural activity and performance level during mobile phone use.

## Introduction

Neurophysiological studies indicate that exposure to electromagnetic fields (EMFs) generated by mobile phone radiation can exert effects on brain activity. Alterations in human EEG induced by mobile phone emitted EMFs have been reported for all common frequency bands (delta, theta, alpha, beta, gamma) of the EEG. A double-blind study on 72 subjects exposed to EMFs for 20 min found an influence on alpha activity under baseline conditions. Alpha activity was significantly reduced under EMF exposure (Perentos et al., 2013). Two studies by Hinrikus et al. (2008) and Suhhova et al. (2013) demonstrated an increase in beta-1 and beta-2 activity, and in contrast to a study by Perentos et al. (2013) observed an increase in alpha activity after EMF exposure. Several studies have shown an increase in alpha-1 and alpha-2 activity in non-REM sleep stages under EMF exposure (Borbély et al., 1999; Huber et al., 2000). Sleep dependent learning processes, such as optimizing motoric skills can be negatively affected by EMF exposure. A significant reduction in motoric skills after a night's sleep under EMF exposure was recorded compared to the control group (Lustenberger et al., 2013). Reduced synaptic plasticity and therefore impaired consolidation by shifting the brain activity have been discussed as the possible mediators for these negative effects of EMF exposure during sleep.

Recent studies have investigated effects of EMF exposure on cognitive functions. For example, EMF exposure over 20 min can impair spatial memory in rats (Jadidi et al., 2007). Similarly, spatial memory processing deficits were found EMF exposed mice (Sienkiewicz et al., 1998). To elucidate the underlying neural mechanisms of the effects of EMF exposure on brain function deficits, studies have shown that EMF exposure can lead to behavioral changes, neurotransmitter releases, and increased blood-brain barrier permeability (Kaviani Mogadam et al., 2004; Luo et al., 2016). Further, research indicates that EMF exposure induced oxidative stress in hippocampus and striatum that might explain impairments in hippocampal-dependent spatial learning (Cui et al., 2012). Alterations in the EEG theta and gamma bands were found to correlate with EMF induced working memory deficits (Zhang et al., 2017).

Neurological and psychiatric disorders can also be aggravated by EMF exposure. Recent research shows interrelations between EMF exposure and sleep disturbances, insomnia, depression and depressive symptoms, restlessness, anxiety, chronic fatigue, dysesthesia, attentional dysfunction, memory changes, headache, dizziness, irritability, loss of appetite, loss of body weight, nausea, skin burning and EEG changes (for a review on the effect of EMFs on these disorders see Pall, 2016). Two longitudinal studies showed that ADHD symptom risk is associated with mobile phone use. Peri- and postnatal cell phone exposure is correlated with behavioral problems and attentional deficits (Divan et al., 2008). Further, increased ADHD symptom risk was associated with mobile phone voice calls but the association was limited to children exposed to relatively high lead (Byun et al., 2013). The pathological shift in brain activity may drive worsening of symptoms and disorders. A good example is the work of Relova et al. (2010) examining epilepsy patients, who reacted with a strong further shift of brain activity to the beta and gamma ranges under exposure to EMFs generated by mobile phones. These EEG high-frequency activities are often associated with seizures.

Children and adolescents seem to be highly sensitive to EMF exposure (for a review see Sage and Burgio, 2018). The study of Croft et al. (2010) recorded a significantly higher increase in EEG alpha activity under EMF exposure to a 2G mobile telephone system than in adult subjects. Recent studies indicate that EMFs can inhibit formation and differentiation of neuronal stem cells during embryonic development and may even negatively affect reproductive and neuronal health of adults, who were prenatally exposed to these fields (see for review Kaplan et al., 2016). Children show a higher absorption of this kind of electromagnetic radiation and an increase in brain temperature was observed in infants (Stankovic et al., 2017).

Summarizing, previous research has shown remarkable effects of EMF exposure on brain activity, cognitive functions, and brain health. The overall shift of brain activity is often associated with different impairments in performance and health.

One technical solution to reduce effects of EMF by mobile phone use is provided by mobile phone chips that are applied to mobile phones or attached to their surfaces. These mobile phone chips are distributed commercially, mostly in European countries and are advertised to have protective effects on the human body against electromagnetic radiation emitted by mobile phones. To date, there are no systematical investigations whether these mobile phone chips have effects on brain activity when exposed to mobile phone-emitted EMFs.

The present study investigated effects of mobile phone chip use on EEG brain activity when participants are exposed to EMFs. Changes in the frequencies of these EEGs can be interpreted as scientific evidence of an indication of the impact of the exposure to EMFs used in mobile communication on brain activity. The EEG waves (in particular the theta, alpha, beta, and gamma bands) can give insights into psychophysiological states such as alertness, awareness, and affection. The frequency range covered by EEG wave measurements can contribute to a better understanding of the physiological (sleep disturbances, states of exhaustion, stress) and psychological changes (enhanced irritability, excitability, lack of concentration) induced by EMF exposure (for a review see Pall, 2016).

One important aspect of this study is the investigation of the effects of mobile phone radiation on brain activity and the ability to concentrate during a cognitive task by measuring the different frequency ranges of the EEG under baseline conditions and during cognitive activity. To date, there are no systematical studies on the effects of mobile phone emitted EMFs under working conditions when the brain is engaged in cognitively demanding tasks as in everyday working settings. Most previous neurophysiological studies have investigated the effects of electromagnetic radiation under resting conditions on brain activity. To test the effects of mobile phone radiation and to evaluate the effectiveness of technical solutions to reduce these effects for working settings, we tested the application of a commercially distributed mobile phone chip under working conditions and measured whether it systematically reduces effects of mobile phone radiation on EEG brain activity.

This study investigated the effects of a mobile phone chip, a placebo chip that used the same raw material as the mobile phone chip, and a control without any chip applied on a smart phone on brain activity under resting conditions, and during an attention test when exposed to EMFs induced by mobile phone radiation. The EEG allows for conclusive assessments of the psychophysiological alertness level as well as of the impact on brain physiology. The data were analyzed by a differentiated assessment of the EEG frequencies in the theta (4–7.5 Hz), alpha (8–13 Hz), beta (14–30 Hz), and gamma (31–70 Hz) bands. This allows interpretations of the different relevancy of the findings in the context of psychophysiological alertness and the concentration and performance ability of the cognitive system determined by the changes in brain activity as recorded by the specific EEG frequency ranges. We hypothesized that the beta and gamma band activity would increase during mobile phone exposure as shown in previous studies (Hinrikus et al., 2008; Perentos et al., 2013; Suhhova et al., 2013). Further, we supposed that application of a mobile phone chip would alter the effects of EMFs emitted by mobile phone radiation on EEG brain activity.

## Materials and methods

### Participants

The present study was performed on 30 healthy subjects in the age of 21–35 years (average age: 25.79 years). All participants were neurologically healthy and had no history of neurological disorders or diseases. All subjects had normal or corrected to normal vision and thus had optimal eyesight. Subjects gave their written consent prior to the study. No subject knew the aim of the study. The subjects were informed on the background and the goal of the study after completion of the study protocol. The experimental procedures were approved by the local ethics committee at the Johannes Gutenberg University of Mainz, Germany. All experimental procedures were in full compliance with the principles outlined in the World Declaration of Helsinki and with national regulations.

### Apparatus

#### Mobile phones and chip application

Three iPhone 5 S were used for this study. The mobile phones differed only in one feature: on one phone, a person independent from the study applied a mobile phone chip (GDM40 15 02 60, Gabriel-Tech, Kelkheim, Germany), on a second phone a placebo chip (GDM40 15 01 60, Gabriel-Tech, Kelkheim, Germany). The third phone was used in the study without a chip, as delivered from the manufacturer (Apple, Cupertino, CA, USA). The testing was performed in a double blind design: neither the caretakers nor the subjects knew which of the three chip condition was applied. The mobile phones were distinguished in the study as “Mobile phone 1” (placebo chip), “Mobile phone 2” (chip), and “Mobile phone 3” (no chip): (1) Mobile 1: iPhone 5 S white, equipped with the placebo chip GDM40 15 01 60, IMEI: 352053069210089, serial number: DX3Q114WFFG9, phone: 0152 0193 7241. (2) Mobile 2: iPhone 5 S white, equipped with the chip GDM40 15 02 60, IMEI: 359266062736925, serial number: DX3QMM6FFG9, phone 0152 0192 5156. (3) Mobile 3; iPhone 5 S white, unchanged phone in original condition as provided, IMEI: 352053068701567, serial number: DX3Q10RVFFG9, phone 0152 0192 0539.

The mobile phones were placed in a specific carrier on a tripod next to the left ear of the subject at a distance of 1.0 cm. A radio was used to induce noise, while measurements of the specific mobile phones (Mobile phone 1, Mobile phone 2, or Mobile phone 3) were carried out during a call received from another mobile phone (iPhone 5 S, Apple, Cupertino, CA, USA). The subjects were provided with earplugs to minimize the background noise and to minimize the acoustic sensory influence on brain activity.

#### Electroencephalogram

Brain activity was recorded via EEG by placing electrodes on the scalp of the subjects. The electrodes served as the interface between the subjects and the equipment for recording the specific brain activity. To allow transmission of the signal from the scalp to the electrodes, an electrolyte was applied (OneStep EEG-Gel®, H+H Medizinprodukte GbR, Münster, Germany). This was composed of sodium and chloride ions. A differential amplifier was used to display the EEG waves. By using the EEG and EMF measures, we were able to record all electric signals of the EEG as well as interfering noise.

Spontaneous EEG brain activity was recorded by the portable 32-channel EEG system Micromed Brain Quick (Venice, Italy) from 19 electrodes placed according to the international 10–20 system with reference to the nose by a sampling rate of 1,024 Hz. During recording the Micromed software system Plus Evolution (Venice, Italy) used a high-pass filter at 0.05 Hz and a low-pass filter at 100 Hz. A bipolar electrooculogram (Micromed, Venice, Italy) for the detection of horizontal and vertical eye movements, and an electromyogram for the recording of electromyographic activity of the neck and shoulder muscles were employed to control for artifacts potentially confounding the EEG measurements. Electromagnetic high-frequency (HF) was continuously recorded as a control variable (ROM Elektronik GmbH, Deisenhausen, Germany).

#### Assessment of EMFs

The measured values for the detection of electromagnetic radiation in the laboratory were all below the threshold values given below during the entire study. For the measurements in the resting state, the subjects were placed on a foam mattress in dorsal position. The d2-R attention test was performed on subjects sitting on an office chair at a desk. The foam mattress was placed at a specifically marked constant position in the laboratory, so that each measurement was performed under exactly the same conditions.

Prior to each measurement, the EMFs in the laboratory, namely the place and the foam mattress on which the subjects were posed, as well as the workplace, where the subject conducted the attention test, were assessed. The alternating electrical field (low frequency, LF), the alternating magnetic field (low frequency, LF), the constant magnetic field (magnetostatics), and the electromagnetic HF were assessed to assure that all measurements were performed under constant conditions and to exclude influences of fluctuations in the external EMFs as well as effects on brain activity. To assess the following five parameters of EMFs the following equipment was used: (1) Alternating electrical field (LF): 3D sensor for alternating electrical fields (LF) designed for a potential independent and a three dimensional measurement (ROM Elektronik GmbH, Deisenhausen, Germany); working range 0–20 V/m, 0–200 V/m, 0–2,000 V/m; frequency range 10 Hz to 400,000 Hz. (2) Alternating magnetic field (LF): 3D sensor for alternating magnetic fields (LF), (Reiner Fauser Elektrotechnik, München, Germany) working range ±0 to ±20,000 nT, frequency range ±5 to ±400,000 Hz. (3) Constant magnetic field (magnetostatics): 3D sensor for constant magnetic fields (ROM Elektronik GmbH, Deisenhausen, Germany); working range ±7 to ± 200,000 nT, frequency range 0–10 Hz. (4) Electromagnetic HF: High-frequency analyzer HFA-3 (ROM Elektronik GmbH, Deisenhausen, Germany); working range 6 to 2,000 m/Vm−0.1 to 10,000 μW/m^2^, (=10 pW/cm^2^ to 1 μW/cm^2^), frequency range 10 MHz to 2.5 GHz. (5) Electromagnetic HF: High-frequency master IV (ROM Elektronik GmbH, Deisenhausen, Germany) working range 6 to 4,400 m/Vm−0.1 to 50,000 μW/m^2^ (10–5 μW/cm^2^) frequency range 1 to 6,000 MHz/1 to 10,000 MHz/ 50 to 3,000 MHz.

The following threshold values for electromagnetic radiation are regarded as harmless according to the German guidelines SBM-2008/IBN for construction biology: (1) Alternating electrical field (LF): <0.3 V/m. (2) Alternating magnetic field (LF): <20 nT. (3) Constant magnetic field: <1,000 nT. (4) Electromagnetic HF: <0.1 μW/m^2^.

### Attention test

**Figure 1:**
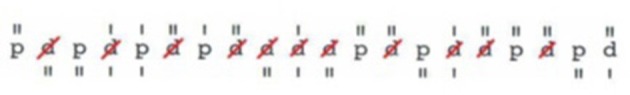
Sample row of the d2-R test.

This study used a diagnostic psychological test procedure, the d2-R attention test (Brickenkamp et al., 2010). The d2-R attention test (d2-R test) is a standardized psychological test method that records the ability to concentrate. A sample row of the d2-R test is depicted in Figure 1. The test is composed of an A4-sized (DIN A4, the German Industrial Norm) page and is filled out with pencil or ballpoint pen. The task of the test person consists of distinguishing specific parts of the test items from others by marking them or scratching them. The test procedure measures the quantity and quality of the work by counting the total number of characters edited and the errors that were made during the process. The test sheet of the d2-R test consists of 14 lines with 57 characters each. The characters are the letters “d” and “p.” They are accompanied by different markers consisting of one to four vertical lines that are positioned over or below the characters. The task of the subjects is to scratch all letters “d” with a total of two lines. Errors consist of missing to scratch a letter “d,” the scratching of the letter “p” and the scratching of a letter “d” with more or less than 2 lines. The test is time limited, so that the subjects have to stop after 5 min. The degree of difficulty of the test is composed of the time limit, and the challenge to discriminate between relevant and irrelevant stimuli.

### Experimental procedure

The study was performed in the laboratories of the Institute of Sport Science of the Johannes Gutenberg University of Mainz (Mainz, Germany). Each participant agreed to participate in three sessions of 60 min for the testing. Three different experimental conditions were tested: Mobile phone 1 = placebo chip, Mobile phone 2 = chip, Mobile phone 3 = no chip. The sequence of the three sessions was randomized to avoid effects due to time, habituation, and routine. The study was performed in a double-blind design. Each experimental session started with the written consent and the description of the purpose of the testing procedure and data collection. Thereafter, another questionnaire was provided to gain insight into demographic data. Further, participants were questioned on neurological impairments, intake of coffee, medication or alcohol, and, questions on the general conditions and sleep quality of the last night before testing. Furthermore, participants completed the standardized questionnaire MDBF (Mehrdimensionaler Befindlichkeitsfragebogen) on their subjective psychophysiological status before and after each experimental condition. After completion of the questionnaires, the apparatus to measure the EEG was applied. All investigations were performed in a laboratory in which no EMFs were measured. Prior to each measurement, the laboratory was scanned for the presence of EMFs using the equipment described above to avoid any interference with the tests. Background EMF noise from experimental mobile phone use was recorded during each measurement to assure constant conditions. A pretest was performed consisting of 2-min EEG measurements under resting conditions with open and closed eyes in dorsal position without a mobile phone application. A 30 min measurement followed applying one of the three mobile phone conditions (Mobile phone 1, Mobile phone 2, or Mobile phone 3). The subjects were placed on a foam mattress with open eyes and a mobile phone was placed at a distance of 1.0 cm from the left ear. For the testing of attentional performance, the attention test (d2-R test) was explained and performed with participants sitting at a desk on an office chair. For each test, the time limit was 5 min. During the attention test, an EEG measurement was performed and the mobile phone was placed near the left ear of the participant. After each exposure to the mobile phone, two measurements were performed with open and closed eyes lasting 2 min each. The testing procedure was performed in a randomized manner in all three specific settings with either Mobile phone 1, Mobile phone 2, or Mobile phone 3 applied.

### Data analysis

#### EEG signal

Continuous frequency analysis also known under the term spectral analysis is of primary importance for the study and will therefore be described in detail below. Spectral analysis depends on the spectra of different EEG sequences that are called compressed spectral arrays (CSA). They demonstrate the distribution of frequencies in the EEG signals and changes of the frequency distribution. By Fourier Transformation, the amplitudes of the EEG waves also contribute to the results. For the handling of the EEG data and their statistical analysis the Matlab based software EEGLAB (Swartz Center for Computational Neuroscience, San Diego, USA) was used. To present the analyzed EEG raw data after the measurement, a multiphase processing of these data with the software was performed. The imported EEG data were processed by an independent component analysis (ICA) and confounding artifacts stemming from eye movements, muscular activity, and EMFs were eliminated manually by the data investigation. The specific power density spectra of the signals were determined by Fast Fourier Transforms for the theta (4–7.5 Hz), alpha (8–13 Hz), beta (14–30 Hz), and gamma (31–70 Hz) bands. The power flux densities of the EMFs were correlated with the calculated power density spectra of the EEGs in chronological sequence.

In a second step, a dipole analysis of the EEG data for the factors chip (chip, placebo chip, no chip) and experimental condition (pretest without mobile phone EMF exposure with open eyes, pretest without mobile phone EMF exposure with closed eyes, mobile phone EMF exposure under resting conditions, mobile phone exposure during the attention test, posttest without mobile phone EMF exposure with open eyes, posttest without mobile phone EMF exposure with closed eyes) was performed. After dissection of the EEG signals by ICA, a three-dimensional head model was calculated, that allowed for the exact localization and representation of the source signals as source equivalent dipoles.

In a third step, a correlation analysis of the continuously recorded electromagnetic HF and the EEG signal was performed. The data of the measurements were imported into the software program Matlab and a correlation analysis with the EEG signal for the theta (4–7.5 Hz), alpha (8–13 Hz), beta (14–30 Hz), and gamma (31–70 Hz) bands was performed.

#### Data on the attention test

Performance in the d2-R attention test was determined for the following parameters: number of edited characters, number of omission errors, number of mix-up errors, and the attention performance score. The number of edited characters indicates the sum of all processed text items and does not consider whether the items were classified correctly or incorrectly. The number of all omission errors includes overlooked or omitted characters. The number of all mix-up errors includes all falsely marked test items. The attention performance score was calculated by subtracting the number of falsely marked test items from the total number of correctly marked characters.

### Statistical analysis

Prior to the study, sample size was calculated using the software GPower 3.0 for matched data (Faul et al., 2007, 2009) to ensure sufficient statistical power for the present study. For the EEG data, 3 × 6 analyses of variance (ANOVAs) for repeated measures were performed for the power density spectra of each EEG frequency band (theta, alpha, beta, gamma). Bonferroni-corrected *post-hoc* tests were performed for the factors chip (chip, placebo chip, no chip) and experimental condition (pretest without mobile phone EMF exposure with open eyes, pretest without mobile phone EMF exposure with closed eyes, mobile phone EMF exposure under resting conditions, mobile phone exposure during the attention test, posttest without mobile phone EMF exposure with open eyes, posttest without mobile phone EMF exposure with closed eyes). To determine the effect size, partial eta-squared was calculated.

The data of EEG dipolar analysis were subjected to ANOVAs with Bonferroni-corrected *post-hoc* tests. Regarding the variable number of activation sources, ANOVAs for repeated measures were performed for the factors chip (chip, placebo chip, no chip), and experimental condition (pretest without mobile phone EMF exposure with open eyes, posttest without mobile phone EMF exposure with closed eyes, mobile phone EMF exposure under resting conditions, mobile phone exposure during the attention test, posttest without mobile phone EMF exposure with open eyes, posttest without mobile phone EMF exposure with closed eyes). To determine the effect size, partial eta-squared was calculated.

For the data of EEG and EMF correlations, the Pearson correlation coefficient was calculated for each EEG frequency band (theta, alpha, beta, gamma). To determine the effect size partial eta-squared was calculated.

For the parameters of the d2-R test (number of all processed characters, number of all omission errors, number of all mix-up errors and overall attention performance), separate ANOVAs for repeated measures were performed. To test the assumption of sphericity, Mauchly's test of sphericity was applied. A Greenhouse-Geisser correction was applied because data did not conform with the assumption of sphericity. In a further step, Bonferroni-corrected *post-hoc* tests were performed. To determine the effect size partial eta-squared was calculated.

A significance level of *p* < 0.05 was set for all statistical analyses.

## Results

### EEG: spontaneous activity

**Figure 2:**
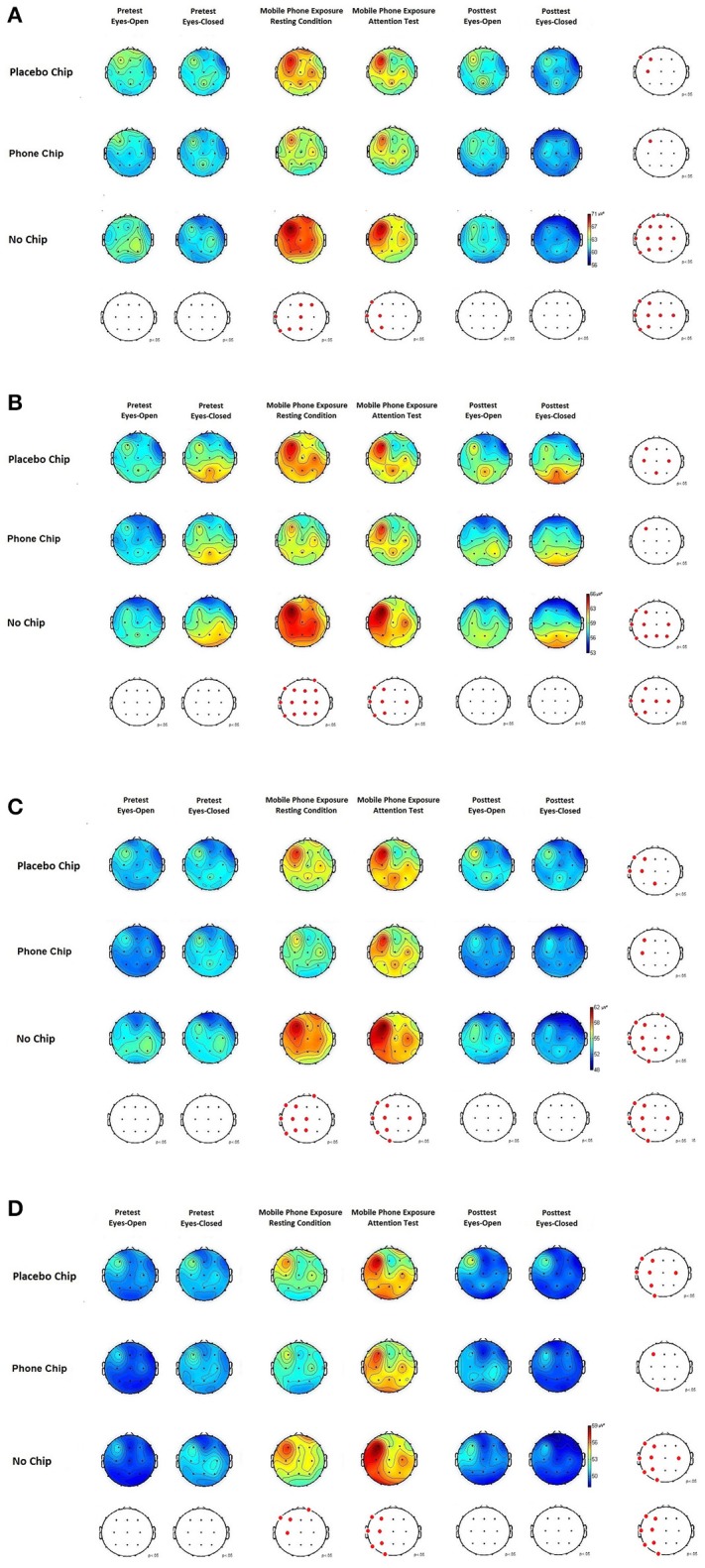
**(A)** EEG theta activity (4–7.5 Hz) before, during, and after application of the placebo chip, the chip and control condition (no chip). **(B)** EEG alpha activity (8–13 Hz) before, during, and after application of the placebo chip, the chip and control condition (no chip). **(C)** EEG beta activity (13–30 Hz) before, during, and after application of the placebo chip, the chip and control condition (no chip). **(D)** EEG gamma activity (31–70 Hz) before, during, and after application of the placebo chip, the chip and control condition (no chip).

The following section presents the results of the EEG measurements under resting conditions and during the attention test. The presentation covers the following frequency ranges: theta range (4–7.5 Hz), alpha range (8–13 Hz), beta range (14–30 Hz), gamma range (31–70 Hz). Figures 2A–D represent the specific frequency ranges and depict the associated brain activity. Results show changes in brain activity in the frequency bands theta, alpha, beta, and gamma during EMF exposure for the placebo chip condition and the no chip condition under resting conditions as well as during the attention test. Application of the chip reduced EMF induced effects on brain activity in all frequency bands during EMF exposure under resting conditions as well as during the attention test.

Spontaneous EEG activity in the theta range is depicted in Figure 2A. The ANOVA for the factor chip showed a significant main effect, *F*_(2, 58)_ = 4.061, *p* = 0.022, partial eta^2^ = 0.13. *Post-hoc* tests with Bonferroni-correction indicated a significant difference between the chip and the placebo chip, *p* = 0.034, and no chip, *p* = 0.015. The ANOVA for the factor experimental condition showed a significant main effect, *F*_(5, 145)_ = 2.931, *p* = 0.015, partial eta^2^ = 0.09. *Post-hoc* tests with Bonferroni-correction revealed significant differences between pretest and mobile phone exposure under resting conditions, *p* = 0.02, and pretest and mobile phone exposition during the attention test, *p* = 0.01. Further, differences were found for comparisons of posttest and mobile phone exposure under resting conditions, *p* = 0.03, and posttest and mobile phone exposure during the attention test, *p* = 0.02. The interaction between the factors chip and experimental condition was significant, *F*_(10, 290)_ = 2.079, *p* = 0.010, partial eta^2^ = 0.08.

Spontaneous EEG activity in the alpha range is depicted in Figure 2B. The ANOVA for the parameter chip revealed a significant main effect, *F*_(2, 58)_ = 4.384, *p* = 0.017, partial eta^2^ = 0.13. *Post-hoc* tests with the Bonferroni-correction indicated a significant difference between the chip as compared to the placebo chip, *p* = 0.031, or no chip, *p* = 0.020. The ANOVA for the factor experimental condition showed a significant main effect, *F*_(5, 145)_ = 3.226, *p* = 0.009, partial eta^2^ = 0.10. *Post-hoc* tests with Bonferroni-correction demonstrated statistical significant differences between pretest and mobile phone exposure under resting conditions, *p* = 0.03, and pretest and mobile phone exposition during the attention test, *p* = 0.03. Further, differences between posttest and mobile phone exposure under resting conditions, *p* = 0.02, and posttest and mobile phone exposure during the attention test, *p* = 0.02, were obtained. The interaction between chip and experimental condition was significant, *F*_(10, 290)_ = 2.319, *p* = 0.012, partial eta^2^ = 0.24.

Spontaneous EEG activity in the beta range is depicted in Figure 2C. The ANOVA for the parameter chip demonstrated a significant main effect, *F*_(2, 58)_ = 3.829, *p* = 0.027, partial eta^2^ = 0.12. *Post-hoc* tests with Bonferroni-correction indicated a significant difference between the chip as compared to the placebo chip, *p* = 0.021, and no chip, *p* = 0.01. The ANOVA for the factor experimental condition showed a significant main effect, *F*_(5, 145)_ = 2.791, *p* = 0.019, partial eta^2^ = 0.09. *Post-hoc* tests with Bonferroni-correction demonstrated a significant difference between pretest and mobile phone exposure under resting conditions, *p* = 0.02, and pretest and mobile phone exposition during the attention test, *p* = 0.009. Further, significant differences were obtained between posttest and mobile phone exposure under resting conditions, *p* = 0.02, and posttest and mobile phone exposure during the attention test, *p* = 0.01. The interaction between chip and experimental condition was significant, *F*_(10, 290)_ = 2.054, *p* = 0.012, partial eta^2^ = 0.06.

Spontaneous EEG activity in the gamma range is depicted in Figure 2D. The ANOVA for the factor chip revealed a significant main effect, *F*_(2, 58)_ = 3.257, *p* = 0.044, partial eta^2^ = 0.10. *Post-hoc* tests with Bonferroni-correction indicated a significant difference between the chip and the placebo chip, *p* = 0.027, and comparison of chip and no chip, *p* = 0.011. The ANOVA for the factor experimental condition showed a significant main effect, *F*_(5, 145)_ = 2.472, *p* = 0.035, partial eta^2^ = 0.08. *Post-hoc* tests with Bonferroni-correction demonstrated significant differences between pretest and mobile phone exposure under resting conditions, *p* = 0.03, pretest and mobile phone exposition during the attention test, *p* = 0.008. Further, differences between posttest and mobile phone exposure in the resting conditions, *p* = 0.03, and posttest and mobile phone exposure during the attention test, *p* = 0.009, were obtained. The interaction between chip and experimental condition was significant, *F*_(10, 290)_ = 2.079, *p* = 0.026, partial eta^2^ = 0.07.

### EEG dipole analysis

**Figure 3:**
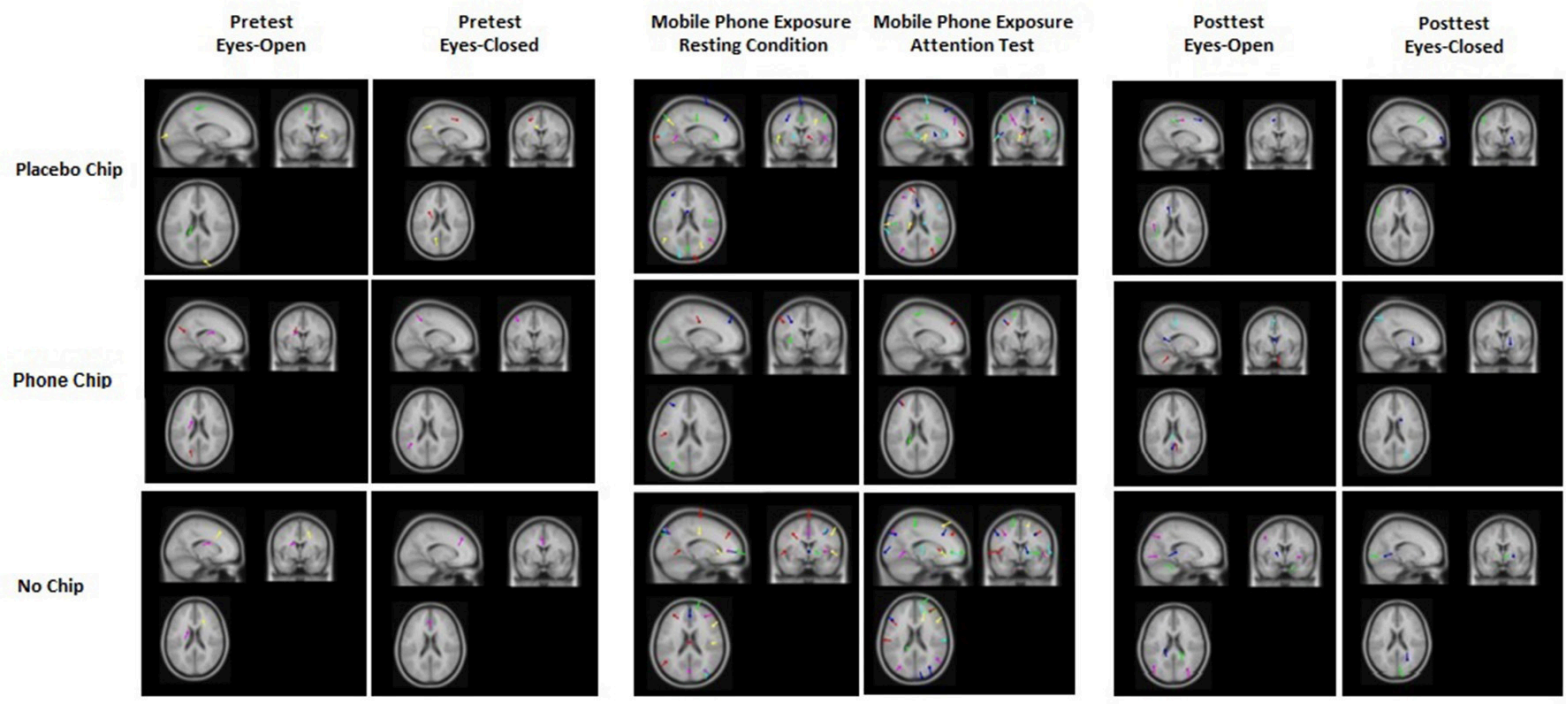
EEG dipole analysis for the placebo chip, the mobile phone chip, and the no chip conditions before, during, and after mobile phone exposure. Results show that more sources of activation were induced in the brain by mobile phone EMF exposure in the placebo chip condition and no chip condition compared to the mobile phone chip condition.

The results of the dipole analysis are depicted in Figure 3. Results showed that more sources of activation in the brain were induced by mobile phone EMF exposure in the placebo chip condition and no chip condition as compared to the mobile phone chip condition. The ANOVA for the parameter chip revealed a significant main effect, *F*_(2, 58)_ = 3.98, *p* = 0.020, partial eta^2^ = 0.15. *Post-hoc* tests with Bonferroni-correction indicated a significant difference between the chip as compared to the placebo chip, *p* = 0.02, or no chip, *p* = 0.02. The ANOVA for the factor experimental condition showed a significant main effect, *F*_(5, 145)_ = 2.81, *p* = 0.017, partial eta^2^ = 0.19. *Post-hoc* tests with Bonferroni-correction demonstrated statistical significant differences between pretest and mobile phone exposure under resting conditions, *p* = 0.03. Further, a difference between pretest and mobile phone exposition during the attention test, *p* = 0.03, and a difference between posttest and mobile phone exposure during the attention test, *p* = 0.03, was shown. Finally, differences between posttest and mobile phone exposure under resting conditions were demonstrated, *p* = 0.04. The interaction between chip and experimental condition was significant, *F*_(10, 290)_ = 2.26, *p* = 0.023, partial eta^2^ = 0.14.

### Correlation of electromagnetic high-frequency and EEG signal

Results suggest medium to strong relations between EMF HF and brain activity. Considering the chip conditions (chip, placebo chip and no chip), the exposure to the chip distinguishes itself from the exposure to the placebo chip and no chip by the lack of significant correlations between EMF HF and the EEG signal in the beta and gamma bands. This indicates that a statistically significant correlation between electromagnetic HF and brain activity in the beta and gamma bands does not occur when the chip is applied.

Correlational analysis indicated highly significant correlations between the electromagnetic HF and EEG activity for the theta range, *r* = 0.63, *p* < 0.001, alpha range, *r* = 0.78, *p* < 0.001, beta range, *r* = 0.83, *p* < 0.001, and gamma range, *r* = 0.75, *p* < 0.001. To consider the influence of the particular chip conditions, the different exposures (chip, placebo chip, no chip) were taken into account and correlations between the different conditions were calculated. After considering the factor chip, highly significant correlations for the placebo chip were demonstrated for the theta range, *r* = 0.74, *p* < 0.001, alpha range, *r* = 0.48, *p* < 0.008, beta range, *r* = 0.86, *p* < 0.001, and for the gamma range, *r* = 0.80, *p* < 0.001. For the chip, highly significant correlations were demonstrated for the theta range, *r* = 0.71, *p* < 0.001, and for the alpha range, *r* = 0.89, *p* < 0.001. The correlations for the beta range, *r* = 0.29, *p* < 0.012, and for the gamma range, *r* = 0.29, *p* < 0.015, were statistically not significant. For the condition no chip, highly significant correlations were demonstrated for the theta range, *r* = 0.82, *p* = 0.001, alpha range, *r* = 0.60, *p* < 0.001, beta range, *r* = 0.71, *p* < 0.001, and for the gamma range, *r* = 0.79, *p* < 0.001.

### Attention test

Means and standard deviations of the d2-R test parameters are depicted in Table 1. The following parameters were taken into account to measure attention: attention performance score, number of edited characters, omission errors, and mix-up errors. Results show lower attentional performance (number of edited characters) during EMF exposure in the placebo chip condition and the no chip condition as compared to the mobile phone chip condition.

**Table 1:** Means and standard deviations of the d2-R test parameters (number of edited characters, mix-up errors, omission errors, attentional performance score).

	**Placebo Chip**		**Phone Chip**		**No Chip**	
	***M***	***SD***	***M***	***SD***	***M***	***SD***
Edited Characters	580.72	102.69	620.26	92.33	570.37	108.75
Mix-up Errors	0.65	1.17	0.42	0.68	0.59	1.32
Omission Errors	5.97	7.24	4.36	3.61	5.88	4.93
Attentional Performance Score	574.14	103.20	615.48	93.74	563.90	107.16

For the attention performance score, the ANOVA indicated a marginal significant main effect for the factor chip, *F*_(2, 58)_ = 2.93, *p* = 0.086, partial eta^2^ = 0.02. *Post-hoc* tests with Bonferroni-correction indicated that exposure with the chip showed significant different results as compared to the placebo chip, *p* = 0.07, and the control condition, *p* = 0.06. For the number of edited characters, the ANOVA for the factor chip indicated a significant main effect, *F*_(2, 58)_ = 3.25, *p* = 0.043, partial eta^2^ = 0.06. *Post-hoc* tests with Bonferroni-correction revealed significant differences comparing the chip and placebo chip, *p* = 0.04, and chip condition with the no chip condition, *p* = 0.04.

## Discussion

The findings of this study demonstrate an effect of EMF exposure by mobile phone use near the head on brain activity. An increase in EEG theta, alpha, beta and gamma activity was observed under placebo and no chip conditions when subjects were exposed to mobile phone emitted EMFs. The results indicate that EMFs emitted by mobile phones can induce local changes in brain activity. The activation primarily of the left side of the brain under exposure to mobile phone-emitted EMFs indicates a relationship between mobile phone-emitted EMF radiation and brain activity, since the test phones were placed at a distance of 1.0 cm from the left ear. Increases in EMF exposure in EEG beta and gamma activity mainly at the left side of the brain were found during the attention test. When the mobile phone chip was applied, a reduction of increases in EEG brain activity was demonstrated compared to the placebo chip and no chip conditions. This was shown for the resting condition, as well as during the attention test.

The increase in brain activity primarily in the frequency ranges beta and gamma indicates an enhanced excitability of the brain under mobile phone associated EMF exposure as shown in the placebo chip or no chip conditions. The findings obtained by dipole analysis confirm the results on EEG brain activity. Dipole analysis showed that more sources of activation in the brain were induced by mobile phone associated EMF exposure in the placebo chip or no chip conditions as compared to the mobile phone chip application. Further, the correlation analysis of continuously recorded HF EMF emission and the EEG signal indicates a strong relationship between HF EMF exposure and brain activity. The mobile phone chip condition is characterized by a lack of correlation in brain activity in the beta and gamma bands as compared to the placebo chip and no chip conditions. This finding indicates that the application of the mobile phone chip can reduce increases in brain activity, mainly in the beta and gamma bands, induced by mobile phone EMF exposure in contrast to the placebo chip and no chip conditions.

The results of the present study mirror findings of previous EEG studies in the research field confirming and extending their observations. Previous research has shown increases in alpha and beta activity (Hinrikus et al., 2008; Suhhova et al., 2013). Further, alterations in the theta and gamma bands were demonstrated confirming results of a previous study by Zhang et al. (2017).

These systematical changes in EEG activity might correlate with impairments in cognitive functions and brain health. Alterations of alpha-1 and alpha-2 activity were previously reported in non-REM sleep stages under EMF exposure (Borbély et al., 1999; Huber et al., 2000). Sleep dependent learning processes that characteristically operate at the theta and alpha bands such as hippocampal theta activity during memory consolidation (e.g., Klimesch et al., 2007) or occurrence of the mu rhythm in motor regions in sleep-dependent optimizing of motoric skills (e.g., Astill et al., 2014) might be negatively affected by EMF exposure. A significant reduction in motoric skills after a night's sleep under EMF exposure was shown compared to a control group (Lustenberger et al., 2013). In this context, it is argued that reduced synaptic plasticity and therefore impaired consolidation by shifting the brain activity are possible mediators for these negative effects of EMF exposure. Further, alterations in the EEG theta and gamma bands were found to correlate with EMF induced working memory deficits (Zhang et al., 2017). The observed changes in EEG frequency bands might be a neural correlate for reduced attentional performance as shown in the present study. Previous work has also demonstrated that EMF induced acute working memory deficits (Zhang et al., 2017) and negative effects on pre-attentive operations (Papageorgiou et al., 2006).

The increase in alpha activity contradicts a finding of a previous study by Perentos et al. (2013) who observed a decrease in alpha activity after EMF exposure. We argue that the difference might result from different experimental setups. Perentos et al. (2013) exposed their subjects for 20 min to EMFs while in the present study and in other work (Hinrikus et al., 2008; Suhhova et al., 2013) subjects were exposed for 35 min or even over longer time intervals, for instance during the whole sleep time (Borbély et al., 1999; Huber et al., 2000). These differences might reflect time-dependent effects of EMF exposure on alpha activity with curvilinear temporal courses of alpha activity during EMF exposure. This assumption needs to be tested in further investigations. A further experimental factor might be the fact that different types of mobile phones were used in previous studies and therefore devices had different transmission powers. Due to technical progress in transmission power the mobile phone used in this study had increased transmission power compared to phones used in past studies. Finally, this might explain why EEG brain activity was altered in all frequency bands. Further studies should investigate the impact of type and transmission power of different mobile phones on brain activity.

Exposure to radio frequency EMFs can significantly increase body and brain temperature (D'Andrea et al., 2007; Stam, 2010; Ohtani et al., 2016). These non-specific thermal effects to EMFs can be harmful as indicated by an induction of heat-shock proteins and heat-shock transcription factors (Ohtani et al., 2016). This stress response is also associated with an increased blood-brain barrier permeability and blood flow that can stimulate brain metabolism and enhance brain activity (Stam, 2010). Sustained exposure can induce a generalized stress response and the enhanced brain temperature is able to evoke a non-specific widespread increase of brain activity over the full range of frequency bands (D'Andrea et al., 2007). This non-specific stress response may be highly detrimental as it is able to enhance higher and lower frequencies simultaneously leading to an overall activation of both, stimulatory and inhibitory neurotransmission at the same time (D'Andrea et al., 2007; Stam, 2010; Ohtani et al., 2016). Since the chip is not a shielding device, it cannot fully prevent these thermal effects on brain activity. Although the new technology was able to significantly reduce the stimulation of brain activity, the harmful thermal effects of EMFs may nevertheless still constitute a significant persisting problem. Thermal effects may be addressed by reducing exposure magnitude and time as they are time- and dose-dependent (D'Andrea et al., 2007; Stam, 2010; Ohtani et al., 2016). The chip can significantly and specifically reduce the activation of brain activity and prevent the shift to higher frequencies as demonstrated conclusively in this study. However, further measures of protection should be considered such as limitation of exposure magnitude and time (D'Andrea et al., 2007; Stam, 2010; Ohtani et al., 2016). Since the mild hyperthermia induced by radio frequency EMFs can cause disruption and even cessation of behavior, this is a further important field of research on safety aspects of exposure (D'Andrea et al., 2007). Our study demonstrated a significant reduction in cognitive ability and performance after exposure to EMFs as the ability to concentrate was impaired. New strategies such as the development of protective chips, a limitation of exposure duration and EMF strength may help to minimize the negative impacts of exposure. The chip was able to reduce the detrimental effects of the exposure to radio frequency EMFs and such devices may be part of a holistic approach in minimizing their negative impacts on brain activity and behavior.

The present findings have important implications for the ergonomic design of working environments where mobile phone use is substantial part of the work. The increase of brain activity in frontal brain areas primarily in the beta and gamma bands indicates that mobile phone associated EMF exposure induces a general psychophysiological activation of the subject. This external stimulation of brain activity induced by mobile phone EMF exposure suggests that it can interfere with cognitive functions associated with the frontal cortex, i.e. attention and working memory (Koivisto et al., 2000b; Papageorgiou et al., 2006; Zhang et al., 2017), and reaction times (Koivisto et al., 2000a). The frontal cortex is a part of the ascending reticular activation system (ARAS) that is responsible for the regulation of the excitability and psychophysiological activation level of humans. Continuous external stimulation, for instance due to mobile phone EMF exposure, leading to a long-term activation of the brain, may result in disorders of the psychophysiological activation level (i.e. overexcitement), problems of subjective sensitivity (i.e. nervousness, irritation) with associated impacts to the mental capability (i.e. concentration problems, weak memory performance), as well as disorders of the regenerative phases of an organism, i.e. during sleep or during regenerative phases during the day (for an overview see Pall, 2016). This is due to changes in the natural composition of the brain activity with its normal distribution to the frequency ranges by this external stimulation. The present study shows that application of the mobile phone chip reduces effects of EMFs on EEG brain activity and attentional performance and therefore contributes to brain health in working environments where mobile phone use is a substantial part of the work.

## Conclusion

The findings of this study are mainly in line with previous studies to date in the field of neuroscience investigating the effects of EMF exposure from mobile phone use on brain activity. Increases in EEG activations were obtained by exposure to EMFs from mobile phone in all tested frequency bands. A reduction of these EMF induced activations is observed when a mobile phone chip is applied, particularly in the high-frequency ranges (beta and gamma bands). The observation is made both in resting as well as in working conditions. A deeper analysis of the EEG signals indicates that when applying the mobile phone chip less activation sources are found in the brain when exposed to mobile phone-emitted EMFs compared to the experimental conditions when a placebo chip is applied, or when no chip is applied. The findings of this study encourage further investigations on the long-term effects of mobile phone chip use in mobile phones in working settings as beneficial effects are shown for the short-term use of mobile phone chips on brain activity.

## Author contributions

All authors listed, have made substantial, direct, and intellectual contribution to the work, and approved it for publication.

### Conflict of interest statement

The authors declare that the research was conducted in the absence of any commercial or financial relationships that could be construed as a potential conflict of interest.
